# The sensitivity of the human thirst response to changes in plasma osmolality: a systematic review

**DOI:** 10.1186/s13741-017-0081-4

**Published:** 2018-01-10

**Authors:** Fintan Hughes, Monty Mythen, Hugh Montgomery

**Affiliations:** Institute for Sport, Exercise and Health, University College London, 170 Tottenham Court Road, London, W1T 7HA UK

**Keywords:** Dehydration, Osmoregulation, Thirst

## Abstract

**Background:**

Dehydration is highly prevalent and is associated with adverse cardiovascular and renal events. Clinical assessment of dehydration lacks sensitivity. Perhaps a patient’s thirst can provide an accurate guide to fluid therapy. This systematic review examines the sensitivity of thirst in responding to changes in plasma osmolality in participants of any age with no condition directly effecting their sense of thirst.

**Methods:**

Medline and EMBASE were searched up to June 2017. Inclusion criteria were all studies reporting the plasma osmolality threshold for the sensation of thirst.

**Results:**

A total of 12 trials were included that assessed thirst intensity on a visual analogue scale, as a function of plasma osmolality (pOsm), and employed linear regression to define the thirst threshold. This included 167 participants, both healthy controls and those with a range of pathologies, with a mean age of 41 (20–78) years.

The value ±95% CI for the pOsm threshold for thirst sensation was found to be 285.23 ± 1.29 mOsm/kg. Above this threshold, thirst intensity as a function of pOsm had a mean ± SEM slope of 0.54 ± 0.07 cm/mOsm/kg. The mean ± 95% CI vasopressin release threshold was very similar to that of thirst, being 284.3 ± 0.71 mOsm/kg.

Heterogeneity across studies can be accounted for by subtle variation in experimental protocol and data handling.

**Conclusion:**

The thresholds for thirst activation and vasopressin release lie in the middle of the normal range of plasma osmolality. Thirst increases linearly as pOsm rises. Thus, osmotically balanced fluid administered as per a patient’s sensation of thirst should result in a plasma osmolality within the normal range. This work received no funding.

## Background

Dehydration is prevalent amongst hospital inpatients. Amongst those aged > 65 years old admitted to UK hospitals, 37% had a plasma osmolality > 300 mOsm/kg on admission, and 62% of these where still afflicted 48 h later (Siervo et al., 2014). While 62% of Scottish stroke patients had a ratio of serum urea: creatinine concentration > 80 mmol/L:μmol/L at some point during hospital admission (Rowat et al., 2012). Over 70% of intensive care unit patients report at least moderately distressing thirst (Puntillo et al., 2010). Dehydration is also highly prevalent outside hospitals; in UK residential homes, measurements of plasma osmolality suggest 46% of residents to be dehydrated (El-Sharkawy et al., 2015).

Such dehydration is not benign: its presence is associated with an increased risk of myocardial infarction (Kloner, 2006), renal calculi (Feehally & Khosravi, 2015), venous thromboembolic disease (Saad et al., 2016) and acute kidney injury (Kanagasundaram, 2015). Meanwhile, dehydration increases pain perception (Farrell et al., 2006) and the associated risk of delirium is comparable with that related to opiate administration (Boettger et al., 2015). As a consequence, dehydration is associated with increased length of hospital stay and greater healthcare costs (Pash et al., 2014; Frangeskou et al., 2015), and preventing dehydration has become a focus of concern for England’s Care Quality Commission (Comission NuTCQ, 2011), patient associations (Association HP, 2010), The British Parliamentary Ombudsman (Parliamentary OH, 2011) and independent inquiries (RF, 2013).

Clinical features of dehydration only appear when fluid losses exceed at least 4–5% of total body water (Mackenzie et al., 1989; Gross et al., 1992) and even then may be variably present or poorly detected. Clinical features of total body water loss include reduced skin turgor, lack of sweat, sunken eyes, and dry mucous membranes, and reflect a reduction in cellular and interstitial water content. Reductions in intravascular volume may be associated with delayed capillary refill time, hypotension (or a postural drop in blood pressure) and tachycardia. However, clinicians are poor at diagnosing dehydration overall when osmolality is used as a gold standard. Diagnosis of dehydration based solely on these signs is unreliable and generally of very low sensitivity (between 0 and 44%) (Fortes et al., n.d.) and poor specificity (Thomas et al., 2004).

Nor can urine or serum osmolality be readily and routinely used to guide hour-by-hour fluid administration. Indeed, no single gold standard test yet exists which can routinely determine hydration status in the clinical environment (Armstrong, 2007). Such factors might account for the huge variation in fluid administration seen in clinical care. By way of example, the volume of fluid administered in the perioperative period was found to vary sixfold between individual doctors at two US institutions irrespective of the patients’ condition (Lilot et al., 2015). Fluid overload can lead to organ dysfunction through the development of tissue oedema. Wound healing can be impaired, and gut function likewise negatively impacted. Oedema increases the diffusion distance of oxygen from capillary to cell, but can also raise the hydrostatic pressure within capsulated organs (such as the kidney) and thus impair tissue perfusion. Pulmonary oedema can also cause a reduction in systemic oxygenation, and increase in end-diastolic pressure can impair subendocardial myocardial perfusion and thus ventricular contractility (Holte et al., 2002).

Physiologically, plasma osmolality (pOsm) is maintained between 275 and 295 mOsm/kg by the combination of thirst sensation and arginine vasopressin release (AVP), stimulated by activation of central osmoreceptors lying outside the blood brain barrier (Baylis & Thompson, 1988). Thirst stimulation will drive fluid consumption to increase total body water, while AVP inserts aquaporins in the collecting duct to promote free-water reabsorption at the nephron via the V2 receptor to prevent further losses from the intravascular space, as well as acting as a potent vasoconstrictor through the V1 receptor (Table 1).

**Table 1:** Summary of included trials investigating the threshold of AVP release and thirst stimulation in response to increasing plasma osmolality

Author (year) (citation)	Age mean	Subject condition	Sample size	Dehydration mechanism	Thirst threshold mean (±SD) mOsm/kg	Relevant findings
(Thompson, Bland et al. 1986)	24.3	Healthy	10	5% NaCl @ 0.06 ml/kg/min for 2 h	281.1 ± 3.2	High individual repeatability of threshold results. Lower threshold found, stimulating thirst before significant dehydration occurs.
(Phillips, Bretherton et al. 1991)	25 69.8	Healthy Young Healthy Elderly	7 7	5% NaCl @ 0.06 ml/kg/min for 2 h	261.0 ± 18.5 276.0 ± 13.2	Elderly show reduced thirst
(Davies, O'Neill et al. 1995)	26.8 70.5	Healthy Young Healthy Elderly	10 10	5% NaCl @ 0.1 ml/kg/min for 2 h	287.5 ± 12.6 292.4 ± 8.5	Thirst threshold is not elevated in healthy elderly, but inter-subject variation is greater. Linear response of thirst to pOsm identified.
(Thompson and Baylis 1987)	29.2 28.6	Healthy Controls Diabetes insipidus	15 14	5% NaCl @ 0.06 ml/kg/min for 2 h	286.3 ± 3.9 286.3 ± 3.9	Diabetes insipidus does not alter thirst or AVP response to pOsm
(Thompson, Davis et al. 1988)	30 29.1	Healthy Controls Type 1 Diabetes	7 7	5% NaCl @ 0.1 ml/kg/min for 2 h vs: Glucose raised from 4 to 20 mmol/l over 2 h	284.7 ± 1.6 287.0 ± 6.9	Oral fluid intake rapidly abolished thirst independent of pOsm. Type 1 Diabetes does not alter thirst and AVP response.
(Thompson, Edwards et al. 1991)	29.6	Healthy controls	7	5% NaCl @ 0.05 ml/kg/min for 2 h	286.5 ± 3.2	No significant difference between thirst and AVP thresholds.
(Thompson, Selby et al. 1991)	34.1	Healthy	16	5% NaCl @ 0.06 ml/kg/min for 2 h	286.3 ± 4.2	Very high 6 month repeatability of AVP and thirst threshold seen within individuals
(Argent, Burrell et al. 1991)	41.1 41.4	Healthy Chronic Kidney Disease	7 8	5% NaCl @ 0.06 ml/kg/min for 2 h	279.4 ± 5.8 281.8 ± 6.8	Threshold of AVP & Thirst are very close in both subject groups
(Phillips, Butler et al. 1994)	41.5	Healthy	8	5% NaCl @ 0.06 ml/kg/min for 2 h vs. 20% Mannitol @ 0.07 ml/kg/min for 2 h	291.0 ± 5.8	5% saline is a more powerful osmotic stimulant than mannitol. The threshold for mannitol is similar but the slope lower
(Martinez-Vea, Garcia et al. 1992)	43.1 55.0	Healthy Controls Chronic Kidney Disease	6 5	5% NaCl @ 0.06 ml/kg/min for 2 h	289.8 ± 8.3 288.9 ± 19.0	High degree of sensitivity and repeatability in individual responses of thirst to osmolality. Thirst unaffected by chronic kidney disease, but dialysis causes a variation.
(Smith, Moore et al. 2004)	51.8	Healthy Controls	8	5% NaCl @ 0.05 ml/kg/min for 2 h	285.9 ± 2.8	Oral fluid intake abolishes osmotically stimulated thirst. Some individuals can lack thirst response.
(McKenna, Morris et al. 1999)	69.8 70.5	Healthy Controls Type 2 Diabetes	7 7	8 h water deprivation	285.5 ± 2.5 283.9 ± 2.0	Osmoregulation of thirst and AVP are normal in Type 2 Diabetes.

Additionally, plasma volume reductions are sensed both directly and indirectly by baroreceptors primarily located in the pulmonary and renal arteries and the atria. Volume depletion also stimulates renin release and thence increased circulating angiotensin II levels which are tightly coupled to increasing thirst (Johnson et al., 1981).

These processes interact; haemodynamic controls amplify the osmotic thirst response. Baroreceptor-signalling mechanisms alter the threshold and sensitivity of both thirst and AVP release to changes in pOsm (Kimura et al., 1976). In hypovolaemic states, the pOsm thresholds for thirst and AVP release are reduced, while the slope of their response to pOsm is increased (Andersson & Rundgren, 1982). This interaction can be explained by the shared vagal and glossopharyngeal pathway from the atria to the supraoptic and paraventricular nuclei of the hypothalamus (Johnson, 2007), which coordinate thirst and AVP release. The osmotic thirst mechanism detects small variations in hydration, while hypovolaemic thirst is specific for large falls in plasma volume of over 8–10% (Kimura et al., 1976).

**Fig. 1:**
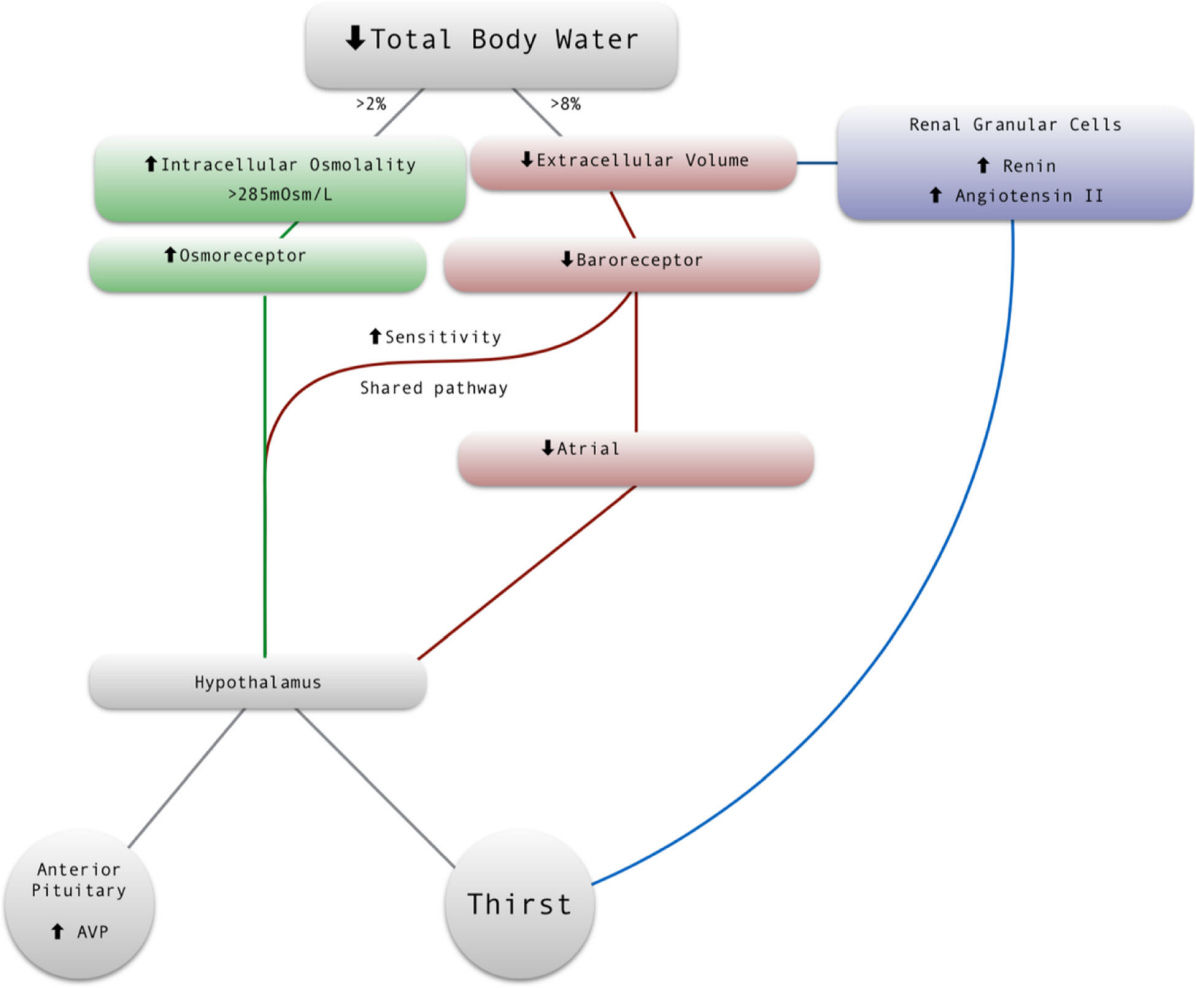
The pathways initiated by total body water deficit leading to the stimulation of the thirst response. Whilst osmoreceptor stimulation is one of three mechanisms which influence the thirst response, it the most sensitive of these, signalling to the hypothalamus after a 2% reduction in total body water. The convergence of baroreceptor and osmoreceptors onto a shared pathway allows for integration of these distinct physiological parameters, such that thirst increases exponentially with large reductions in extracellular volume

Given the integrative nature of these homeostatic mechanisms, could a patient’s own subjective sense of thirst be a better guide to the need for further hydration than our current clinical assessment? The degree to which clinicians include assessment of thirst when considering fluid prescription is not known. Anecdotal evidence suggests that some more experienced clinicians may do so. However, the value of this may be influenced by the degree to which thirst reflects a dehydration-related rise in serum osmolality. Perhaps thirst is one of the few sensitive symptoms of underlying reductions in total body water, and should prompt further clinical and biochemical investigation. If so, this might guide fluid administration in hospitalised patients, fluid being delivered until thirst is no longer present. However, before such practice can be recommended, it is essential to quantify the diagnostic accuracy of thirst so as not to pose a risk of iatrogenic dehydration or fluid overload. To explore the feasibility of this approach, we performed a systematic review to determine the value of plasma osmolality associated with developing a sense of thirst, how this relates to age and gender, and those factors which might influence thirst in hospitalised patients (Fig. 1).

## Methods

Medline and EMBASE were searched (up to June 1, 2017) for human trials in all languages for the combined terms ‘thirst’ AND ‘osmolality’ AND ‘threshold’, the bibliographies of extracted papers were also searched for relevant articles.

Included studies were those that reported the plasma osmolality threshold, in dehydrated subjects, for the sensation of thirst, as measured on a visual analogue scale. Full journal publication was required. Participants were of any age, with no condition directly effecting their sense of thirst. The types of interventions included were any which simulated or induced dehydration by increasing plasma osmolality.

Trials included were required to assess thirst intensity as a function of plasma osmolality, and employ linear regression to define the threshold value of pOsm for the sensation of thirst. This allows for both averaging of the thirst response across a range of dehydration severity and identifies the threshold more precisely than a subject reporting their onset of thirst.

Tabulated data were extracted from the included trials directly into spreadsheets to become the input for our statistical analysis. The primary variable sought was the pOsm threshold for thirst. Secondary variables, analysed where available, included pOsm threshold for the release of arginine vasopressin (AVP), the rates at which thirst score and AVP concentration varied with increasing pOsm, and linear correlation coefficients of both the thirst and AVP response to pOsm. Each value extracted was accompanied by a measure of variation, being either standard deviation, standard error or 95% confidence intervals. Data relating to the rate of increase in thirst were normalised to account for differences in size of the visual analogue thirst scales between the studies.

The factors affecting the sensitivity of thirst are poorly understood and no fixed effects can be assumed for the individuals in these studies. As the cohorts studied are not identical, some measurement errors exist and the results are intended to be generalised, a DerSimonian and Laird random effects model was employed with the R metafor package (Figure 3), which also provided an inconsistency metric for heterogeneity.

Whilst the synthesis of regression slopes is the topic of much discussion, this complexity arises from the combination of dissimilar studies and resulting nonequivalence of metrics (Becker & Wu, 2007). In the case of each of the studies included in this analysis, however, which employ a univariate approach, the two parameters, pOsm and thirst intensity or AVP concentration were measured in a comparable manner. As such it is appropriate to synthesise regression slopes using an arithmetic mean.

Papers varied in their reporting of either standard error of the mean, standard deviation or 95% confidence intervals; these were converted into variances and combined by Satterthwaite approximation (Satterthwaite, 1946), due to the non-equal variances within each patient cohort, to produce a composite variance for each secondary parameter.

Studies were subjectively assessed for sources of bias, with respect to selection of participants, performance bias, detection bias, and attrition bias.

Additional analysis was performed on the thirst thresholds reported in the subgroup of female trials. A paired *t* test was used to assess the mean difference between follicular and luteal phases within the menstrual cycle across four studies. These paired values were also combined to give a mean value for the female thirst threshold.

## Results

One-hundred seventeen studies identified by our search strategy were screened and assessed, with 22 deemed eligible. Two studies were excluded that reported thirst threshold based on a single data point, not through least squared regression. A further study which did not include distribution data for each outcome was also excluded.

In women, there is known variation in thirst between different hormonal states. Seven studies investigating the effects of hormonal variation on thirst were excluded from the analysis and reviewed separately. The values reported in these trials are for specific cohorts of subjects across a range of hormonal states, which are either pharmacologically or pathologically induced, or related to the physiological variations at discrete time points in pregnancy and the menstrual cycle. Thus, the distribution of hormonal states in the pooled cohort from these studies is not representative of the normal population.

**Fig. 2:**
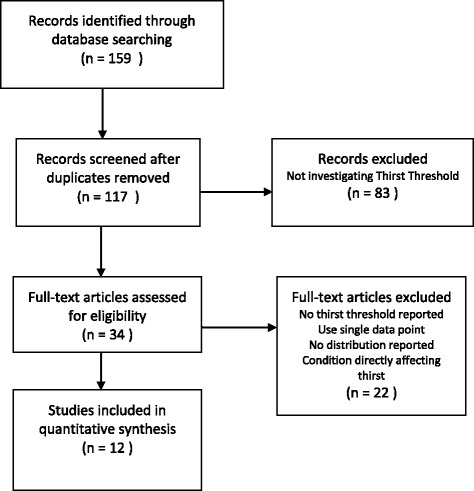
Flowchart showing articles retrieved and considered at each stage of the review process

In the primary analysis, a total of 12 trials were included. Cohorts of patients with a significant disturbance in fluid balance (e.g., dialysis causing > 5% weight gain, compulsive water drinking, syndrome of inappropriate antidiuretic hormone secretion and those following hyperosmotic non-ketotic coma) were excluded from the analysis (Fig. 2).

### Study characteristics

Included studies involved 167 participants with a mean age of 41, ranging from 20 to 78 years. These included both healthy controls and those with a range of pathologies: diabetes insipidus, diabetes mellitus type 1 and 2, and chronic kidney disease.

During the period prior to each trial, the intake of participants was standardised in different ways, ranging from 12 h of fasting with free water intake, to avoidance of caffeine on the morning of the trial. One trial imposed a standard protocol for fluid consumption in the 12 h prior to the experiment.

In all but one trial, which employed water deprivation, subjects rested in a recumbent or supine position for 15 to 60 min before intravenous cannulae were placed into each antecubital fossa.

One cannula was used for the infusion with blood samples drawn from the opposite arm. In all but one trial, pOsm was determined by freezing point depression.

All studies of high enough quality to include in our analysis followed the same general experimental design; defining thirst score as a function of plasma osmolality, they studied each participant’s data over the course of a progressive rise in plasma osmolality. Seven trials administered a peripheral intravenous infusion of hypertonic saline at a rate of 0.06 ml/kg/min over 2 h (Thompson et al., 1986; Phillips et al., 1991; Thompson & Baylis, 1987; Thompson et al., 1991; Argent et al., 1991; Phillips et al., 1994; Martinez-Vea et al., 1992), with four others using rates of 0.1 (Davies et al., 1995; Thompson et al., 1988) and 0.05 ml/kg/min (Thompson et al., 1991; Smith et al., 2004). One trial compared saline infusion to a 2 h 20% mannitol infusion at 0.07 ml/kg/min (Phillips et al., 1994), and another compared hypertonic saline to a steady infusion of hypertonic D-glucose, at a rate adjusted to each participant, sufficient to raise plasma glucose from 4 to 5 mmol/L to 20 mmol/L over 2 h (Thompson et al., 1988), another enforced 8 h of fluid restriction as a method of dehydration (McKenna et al., 1999).

These protocols led to an increase in pOsm of approximately 20 mOsm/kg, from starting values of between 276 and 290 mOsm/kg.

Trial participants reported thirst intensity (using a visual analogue scale, ranging from 100 mm long to 180 mm long) over the course of the dehydration challenge. Participants would mark the intensity of their subjective sense of thirst on these scales, and the distance from the zero point defined the degree of thirst intensity. None of the scales were graduated, but some included text at the extremes of the scale, indicating ‘not thirsty’ or ‘very thirsty’. Linear regression analysis was applied to a series of subjective thirst scores taken throughout the course of the dehydration challenge, plotted against measurements of plasma osmolality. These linear regression models were then used by each study to determine primary outcome, the minimum value of plasma osmolality required for the sensation of thirst. This was achieved by calculating the abscissal intercept, which is the value of plasma osmolality above which thirst starts to increase. Secondary outcomes reported in several studies were the pOsm threshold for AVP release, the rate of increase of both thirst and AVP concentration with pOsm and the correlation coefficients of each subject to the linear regression model.

Four of these trials investigating female thirst were conducted in much the same way as those in males, reporting thirst scores on a visual analogue scale to changes in plasma osmolality due to hypertonic saline infusion or fluid restriction (Evbuomwan et al., 2001; Stachenfeld et al., 1999; Stachenfeld & Keefe, 2002; Calzone et al., 2001). The other three studies, whilst similar in design, only reported single values for thirst threshold; whilst this technique consistently reports higher thirst thresholds than those found through least squared regression, these data are still relevant for comparisons within each trial (Davison et al., 1988; Thompson et al., 1988; Spruce et al., 1985).

### Data analysis results

Data on the thirst threshold were available in all included trials. The value ±95% C.I. for the pOsm threshold for thirst sensation was found to be 285.23 ± 1.29 mOsm/kg (*n* = 167). There was evidence of significant heterogeneity between studies (*I*^2^ = 0.73, *τ* = 4.53). None of the secondary outcome measures were present in all studies. Above this threshold, thirst intensity as a function of pOsm was found to have a mean ± SEM slope of 0.54 ± 0.07 cm/mOsm/kg (*n* = 143). The mean correlation coefficient of each individual linear regression was 0.91 (*n* = 120), indicating that above the threshold for sensation, the increase in thirst with pOsm is linear.

**Fig. 3:**
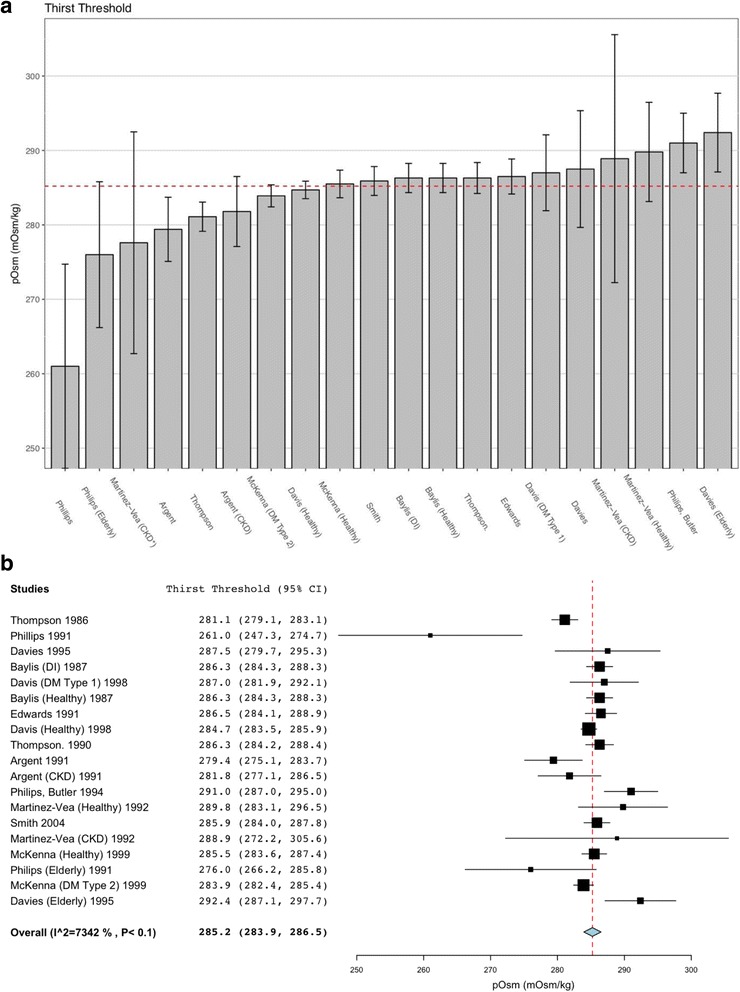
Comparison of pOsm thirst threshold across the 19 subject cohorts of the 12 studies, with 95% confidence intervals. The mean value 285.2 mOsm/kg is displayed in red

Eight studies also examined the threshold of AVP release in response to changes in pOsm. The mean ± 95% C.I. AVP release threshold was very similar to that of thirst, being 284.3 ± 0.71 mOsm/kg (*n* = 150). Above this threshold, AVP release was also linear, as shown by a mean regression coefficient of 0.91 (*n* = 57), and had a mean ± SEM slope of 0.35 ± 0.09 pmol/mOsm (*n* = 72) (Fig. 3).

### Female thirst data

The pooled data from four studies (Stachenfeld et al., 1999; Calzone et al., 2001; Thompson et al., 1988; Spruce et al., 1985) in women demonstrated the mean reduction in thirst threshold in the luteal phase to be 3.1 mOsm/kg (*p* < 0.001). Averaging the control values for both follicular and luteal phases returns a threshold of 282.7 mOsm/kg (*n* = 26).

Combined administration of oestrogen and progesterone produced a threshold 4 mOsm/kg lower than administration of oestrogen alone (Stachenfeld & Keefe, 2002), and 2–4 mOsm/kg lower than that of progesterone alone (Stachenfeld et al., 1999; Calzone et al., 2001). Female thirst is further affected by pregnancy, one study showing that thirst threshold consistently falls from a pre-conception value of 285.5 to 281.5, 277.5, and 275.5 mOsm/kg across the gestation period, before returning to the starting value (Davison et al., 1988) by 10 weeks postpartum.

No bias likely to have significantly affected the cumulative results was identified. Only 3 of the 12 studies employed randomisation to the selection of participant or their allocation into groups. In the other studies, allocation into experimental or control arm was determined by participant factors (either age or pathology). Double blinded control infusions of physiological saline were employed in two studies, which caused no alteration in thirst sensation. Trials were supported either by national funding bodies or medical research charities, except for three studies which did not report the source of their funding. In no case was bias considered a likely contributing factor to heterogeneity in reported thirst threshold.

## Discussion

### Sources of heterogeneity

Whilst the heterogeneity seen in our primary outcome across studies was considerable, it is not indicative of the degree of thirst threshold variation within the population. Instead, subtle variation in experimental protocol and data handling can account for much of the heterogeneity.

Both studies of chronic kidney disease report adjusted pOsm values to facilitate comparison between controls and subjects; in the case of Argent (Argent et al., 1991), these were corrected to a urea concentration of 0, which will give an artificially low thirst threshold; conversely Martinez-vea corrected pOsm upwards to a standardised blood urea nitrogen of 10 mg/100 ml, accounting for the higher threshold seen (Martinez-Vea et al., 1992). Notably, this was the only study not to impose limits on subjects’ pre-trial intake.

The elevated threshold in the 1994 trial by Philips and Butler can be explained by their use of two-segment piecewise linear regression. From their assumption that the thirst response is parabolic, they forced a turning point into their regression model and defined this as the threshold (Phillips et al., 1994). The experiment raised pOsm from 287 to 304 mOsm/kg, which by necessity will produce a threshold within this range, using this segmented regression.

Davies’ 1995 study combined data points from both the saline infusion and a subsequent period of water drinking into their calculation of the thirst threshold. As stimulation of peripheral oropharyngeal osmoreceptors leads to inhibition of thirst, this method produces lower thirst scores and therefore reports a higher threshold for thirst (Davies et al., 1995).

The most significant outlier is Philips’ 1991 study. This study is unique in its measurement of pOsm, determined by a vapour pressure osmometer, based on dew point depression, which has a tendency to produce lower results for pOsm (Koumantakis & Wyndham, 1989). Indeed, even the starting values of pOsm reported for participants was 9 mOsm/kg lower in this study than any other. Additionally, subjects were asked to score not only their thirst, but also the dryness of their mouth, which likely combined to increase ratings on the visual analogue scale.

Whilst values for the threshold fall within a narrow range, the relationship of thirst intensity to rising osmolality shows marked inter-individual variation. Importantly the variation seen in this threshold is mainly derived from inter-subject variability, with a very high degree of repeatability being observed for each individual participant (Thompson et al., 1986). Indeed, when the same eight subjects underwent a repeat saline infusion after 6 months, the composite variances in values for both thirst and AVP thresholds of each individual subject compared to the initial values, were found to be 0.6% (Thompson et al., 1991). It thus appears that each person’s thirst response remains a consistent indicator of his level of dehydration. Perhaps this ‘individual threshold’ is associated with ‘healthy hydration’ for that subject, and the stimulation of thirst and AVP release act to return a subject to this set point of hydration.

There exists some disagreement in the literature regarding whether AVP release shares the same response to pOsm as the thirst. Our analysis demonstrates a 0.9 mOsm/kg difference between these two thresholds. This result is consistent with the findings of Thompson et al.’s 1986 study of healthy men, which concluded that AVP release and thirst sensation have a shared threshold (Thompson et al., 1986). However, such a result conflicts with data reported by others, Robertson suggesting that the thirst threshold was consistently higher than the threshold for AVP release (Robertson, 1984).

### Variations in the thirst response seen in the elderly

It is possible that the thirst and/or AVP thresholds change with age. Philips et al. described a reduction in thirst response in elderly men despite normal AVP secretion (Phillips et al., 1991). In contrast, however, Davies et al. found no significant difference in the slope or threshold of thirst response to pOsm between young (< 40) and ‘healthy’ elderly (> 70) participants. They found that age only affected thirst response to rapidly changing pOsm (Arnaud, 2003). With age, the inter- and intra-subject variability of thirst increases, whereas that of AVP release does not (Phillips et al., 1991; Davies et al., 1995). This variability is seen in response to differences in experimental conditions, such as altered rate of saline infusion; it is possible that the subjects undergoing healthy ageing, described in Davies et al.’s work, are not representative of the cross section of the elderly population found in hospital, many of whom may have multiple morbidities. It is important to recognise that much of the basis for the widely held assumption that thirst is diminished in the elderly is based on evidence from hospitalised older patients (Kenney & Chiu, 2001). Yet, in the carefully controlled experimental conditions utilised in the reviewed studies, thirst sensing did not appear altered by age. However, it does appear that elderly subjects do not drink as much as younger subjects upon dehydration and similar AVP responses (Phillips et al., 1984).

In any work regarding thirst, it is worth noting the important role played by peripheral osmoreceptors, primarily those which generate afferent signals from the oropharyngeal region. These produce a marked and sudden inhibition of AVP and thirst in response to drinking, which appears greater than can be ascribed to any change in systemic osmolality and which antecedes such osmolality changes (Figaro & Mack, 1997). As such, access to oral fluids may reduce the thirst score associated with any rise in plasma osmolality, and may thus increase experimental thirst threshold values and thirst-osmolar gradient. Most notably, those studies reporting a reduction of thirst sensing in the elderly have examined this question in relation to oral fluid intake (Mack et al., 1994; Miescher & Fortney, 1989; Phillips et al., 1984; Takamata et al., 1999). It remains to be seen whether this finding is consistent in the case of intravenous rather than oral rehydration, although it is possible that oropharyngeal suppression of osmotic thirst is greater in the elderly. If so, thirst will remain a good guide to intravenous fluid management of dehydration in both the young and old. The thirst of elderly subjects’ is less sensitive to isolated experimental variations in plasma volume (Stachenfeld et al., 1997). However, given the interdependence of osmotic and haemodynamic thirst mechanisms, future studies should focus on the combined hypertonic-hypovolaemic dehydration typical in clinical practice.

### Variations in the thirst response seen in women

Over the course of the menstrual cycle, there is marked variation in the levels of circulating oestrogen and progesterone. Whist the follicular phase is dominated by an oestrogen spike at day 12, the luteal phase sees high levels of both oestrogen and progesterone peaking at day 22. These hormones are known to influence the thirst response by several possible mechanisms of actions. AVP’s effect at the kidneys is modulated by oestrogen, whilst α and β oestrogen receptors are found in AVP neurons of the hypothalamus, and along both osmoreceptor and baroreceptor pathways, controlling AVP release (Sladek & Somponpun, 2008).

Whilst the combination of oestrogen and progesterone produce a threshold-lowering effect, neither hormone in isolation is responsible for this effect. Combined administration of oestrogen and progesterone produced a greater reduction in threshold than either hormone in isolation.

### Application to a hospitalised population

‘Thirst threshold’ appears remarkably consistent in the healthy, suggesting that patient thirst may be a useful guide to fluid administration in such individuals. However, this may not be so in hospitalised patients: haemodialysis (Martinez-Vea et al., 1992) and opioid administration, for instance, might perhaps both be associated with increases in thirst (Stotts et al., 2015). Morphine use was associated with a dry mouth in 47% of intensive care patients, with 3% of fentanyl users afflicted (Wiffen et al., 2014). Whilst causation was unproven in this study, high doses of morphine may stimulate drinking whilst low doses may inhibit thirst (Vokes, 1987). The specific nature of thirst response to opioids is thus hypothesised to depend on dose administered and the receptor being targeted, with μ_2_, δ_1_, and κ receptors modulating angiotensin II-induced thirst (Wilson et al., 1999). Likewise, insulin and epinephrine appear to stimulate thirst, whilst norepinephrine, haloperidol, and glucocorticoids may be inhibitory (Vokes, 1987). Thirst is shown to increase the intensity of contemporaneous pain whilst no changes were seen in thirst rating in response to pain (Farrell et al., 2006). Whether such influences are of clinical relevance is not known.

Interestingly, diabetes mellitus, a condition often associated with disturbances in thirst, was found to have no influence on thirst threshold (McKenna et al., 1999). The administration of a glucose infusion, raising blood glucose levels to 20 mmol/L did not influence either thirst or AVP levels. This suggests that the polydipsia experienced in uncontrolled diabetes is a consequence of hypovolaemia induced by polyuria, rather than disturbances in plasma osmolality (Thompson et al., 1988).

## Conclusion

Thirst and AVP respond to increases is plasma osmolality in unison, acting as the primary homeostatic mechanism of body water regulation. Across a range of physiological states including young healthy males, in patients with chronic kidney failure, and in subjects over the age of 70 and participants with both types 1 & type 2 diabetes, values for the pOsm threshold of thirst response fell within a narrow range.

Our analysis has demonstrated that across a diverse population of participants the thresholds for thirst activation and AVP release are exactly in the middle of the normal range of plasma osmolality. Both rise linearly with pOsm, intensifying the mechanisms acting to reduce pOsm, either by stimulating water consumption or stimulating water retention at the nephron. It can be assumed that the presence of the symptom of thirst likely indicates a hyperosmotic state. Thus, clinicians might use the symptom of thirst to screen for the need for further assessment of hydration status. This clinical examination can be facilitated by requesting a measurement of plasma osmolality, or alternatively through simple calculations from the patient’s existing biochemistry data. The best formula for doing do being: plasma osmolarity (in mmol/L) = 1.86 × (Na^+^+ K^+^) + 1.15 × [glucose] + [urea] + 14 (Hooper et al., 2015).

Whilst the exact levels of plasma osmolality at which thirst can be sensed vary between individuals, intra-individual measures are highly consistent and reproducible. Thirst may be a more sensitive indicator of dehydration than a clinician’s assessment of physical signs. As such, our patient’s feedback as to their sense of thirst could hold the essential information needed to accurately manage their fluid administration for both pre- and post- operative optimisation.

These existing trials are limited in their scope, in that they artificially induced a hyperosmolar state in small and restrictive cohorts. The time consuming and invasive nature of the experimental designs used limit the cohort size to an extent. The volume expansion caused by the administration of fluids may also interfere with the sensation of thirst as baroreceptors stimulate atrial natriuretic peptide release, inhibiting thirst. The lack of hypovolaemia accompanying the hyperosmolar plasma excludes a true investigation of the thirst response which increases exponentially as plasma volume falls. The model of thirst examined by these studies remains representative of mild to moderate degrees of dehydration, more relevant to perioperative optimisation. These studies remain of value in their assessment of a fundamental physiological parameter, but further investigations in surgical patients throughout the perioperative period would be of value. Our analysis of these trials was limited by their age, and the associated difficulty in attaining the raw data. We rely on the data analysis of each individual author and our analysis inherits a degree of heterogeneity as a result. Our review is further limited in that it does not include hospitalised patients.

Fluid management is an area that still requires significant improvements, yet the thirst response has been overlooked as an area of research over the last decade. A full population based model of thirst is still lacking, and we would advocate further research in this area. Firstly, the true prevalence of elevated plasma osmolarity might be systematically sought in patient electronic records. Secondly, the ability of a variety of clinicians to identify varying degrees of dehydration should be assessed across a range of severities. All such findings might be related to the quantified thirst intensity as perceived by patients. This may be facilitated by the prospective inclusion of thirst intensity into clinical research databases as a patient reported outcome measure. Factors influencing that thirst response (such as opioid analgaesic use, anticholinergic drug effects, obtundation, use of dry inhaled gases) might also be systematically sought. The full description of those factors that affect thirst in the hospitalised patient, by way of large-scale observation studies would allow for the design of individualised thirst-based fluid administration systems, specific to a patient’s pathology and medications. Furthermore, explicit investigation of whether thirst might be used to guide or trigger the administration of intravenous fluid boluses in the clinical setting is warranted. Clinical trials might asses the ability of healthy volunteers and of patients to better guide their intravenous fluid therapy.

## Abbreviations

AVP: Arginine vasopressin
pOsm: Plasma osmolality
SEM: Standard error in the mean
